# 2418 Post-traumatic stress symptoms in caregivers of pediatric hydrocephalus population

**DOI:** 10.1017/cts.2018.299

**Published:** 2018-11-21

**Authors:** Kathrin Zimmerman, Alexandra Cutillo, Laura Dreer, Anastasia Arynchyna, Brandon G. Rocque

**Affiliations:** University of Alabama at Birmingham

## Abstract

OBJECTIVES/SPECIFIC AIMS: The goal of this study is to characterize traumatic events and post-traumatic stress symptom severity experienced by caregivers of children with hydrocephalus. Results will eventually be evaluated and compared with demographic and medical characteristics. This study is part of a larger research project that aims to (1) determine the prevalence and risk factors for post-traumatic stress symptoms in pediatric hydrocephalus patients and their caregivers; (2) develop a targeted intervention to mitigate its effects and pilot test the intervention. METHODS/STUDY POPULATION: Caregivers of children with hydrocephalus that have received surgical treatment (CSF shunt or ETV/CPC) were enrolled during routine follow up visit in a pediatric neurosurgery clinic. Caregivers completed the PTSD Checklist for DSM-5 (PCL-5), a 20-item self-report measure that assesses the presence and severity of post-traumatic stress disorder (PTSD) symptoms. RESULTS/ANTICIPATED RESULTS: Participant responses (n=56) revealed that 57.14% of caregivers indicated that their most traumatic event was directly related to their child’s medical condition. In total, 23.21% of caregivers did not specify their most traumatic event and 1.79% of caregivers indicated that they had never experienced a traumatic event. Median Total Symptom Severity Score was 11 (mean: 15.32±14.92), and scores ranged from 0 to 67; 32.14% of caregivers scored 19 or greater, and 16.07% of caregivers scored 33 or greater, a value suggestive of a provisional diagnosis of PTSD. Severity scores by DSM-V clusters were as follows: cluster B—intrusion symptoms (mean: 4.91±4.77, median: 4, range: 0–20), cluster C—avoidance symptoms (mean: 1.27±1.87, median: 0.5, range: 0–8), cluster D—negative alterations in cognition and mood (mean: 4.86±6.07, median: 2, range: 0–22), and cluster E—alterations in arousal and reactivity (mean: 4.29±4.07, median: 3, range: 0–17). DISCUSSION/SIGNIFICANCE OF IMPACT: Preliminary results from this study indicate that post-traumatic stress symptoms are prevalent among caregivers of children with hydrocephalus. These results suggest that psychosocial issues such as PTSS may be a significant problem in need of treatment, that is not traditionally addressed as part of routine care for families of children with hydrocephalus. Characterizing post-traumatic stress symptoms in this population sets the foundation for the development of screening and treatment protocols for post-traumatic stress symptoms in caregivers of children with hydrocephalus. This study is the first step towards fundamentally improving routine clinical care and quality of life for patients with hydrocephalus and their caregivers by understanding and addressing the effects of traumatic stress.

